# Fournier’s Gangrene Severity Index and Charlson Comorbidity Index Predict Mortality, Intensive Care Unit Admission, and Length of Hospital Stay in Patients With Fournier’s Gangrene: An Analysis of 264 Consecutive Patients

**DOI:** 10.7759/cureus.75281

**Published:** 2024-12-07

**Authors:** Nicholas Hauser, Chenlin Huang, Girija Vaidya, Timothy Chrusciel, Krithika Narayana Kumarasan, John Culhane, Sameer Siddiqui

**Affiliations:** 1 Urology, Saint Louis University School of Medicine, Saint Louis, USA; 2 Urology, Baylor College of Medicine, Houston, USA; 3 Data Analytics, Saint Louis University School of Medicine, Saint Louis, USA; 4 Surgery, Saint Louis University, Saint Louis, USA; 5 Urology, SSM Health Saint Louis University Hospital, Saint Louis, USA

**Keywords:** charlson comorbidity index, fournier gangrene, fournier gangrene severity index, necrotizing fasciitis, urology

## Abstract

Introduction

Fournier’s gangrene (FG) is a rapidly progressing necrotizing fasciitis. The Fournier's Gangrene Severity Index (FGSI), in conjunction with the Charlson Comorbidity Index (CCI), has been used as a mortality predictor during hospitalization. Patients with diabetes have also been shown to be at an increased risk for the development of FG. This study explores the potential of using FGSI, CCI, and patient diabetes status in predicting more extensive patient outcomes, including intensive care unit (ICU) admission and length of hospital stay.

Methods

From 2013 to 2023, inpatient admissions with the diagnosis of FG were selected from the EPIC Clarity database of the Sisters of St. Mary hospital system. Patient demographics, ICU admission, length of hospital stay, medical comorbidities, and calculated FGSI and CCI scores were extracted from selected admissions. Logistic regression and generalized linear regression models were performed for regression analysis.

Results

A total of 264 patients who met our inclusion criteria were admitted for FG from 2013 to 2023. The patient population was all male with a mean age of 54.7 years. Of the cohort, 127 patients (48.1%) required ICU care, and 44 patients (16.7%) died during admission. Of the 164 patients (62.1%) diagnosed with diabetes, 13 were taking sodium-glucose cotransporter 2 (SGLT-2) inhibitors prior to admission, and 121 were insulin-dependent. No relationship could be found between the use of SGLT-2 inhibitors or insulin and patient outcome. For every one-unit increase in FGSI score, the odds of mortality increased by 1.14 times, the odds of being admitted to the ICU increased by 1.3 times, and the length of hospital stay increased by 1.1 days (p<0.0001). For every one-unit increase in CCI score, the odds of mortality increase by 1.2 times (p=0.003), and the length of hospital stay increased by 0.9 days (p=0.04).

Conclusions

Insulin-dependent diabetes and the use of SGLT-2 inhibitors are not risk factors for more severe FG. FGSI and CCI scores may be used to predict patient outcomes beyond mortality, including ICU admission and length of hospital stay.

## Introduction

Fournier’s gangrene (FG) is a necrotizing infection involving the lower abdominal, genital, and perineal areas. The disease is the result of a bacterial infection of the underlying Dartos, Colles, or Scarpa’s fascia, which causes severe inflammation of the deep and superficial tissue that rapidly spreads throughout the region leading to skin breakdown [1]. The initial infection can have a multitude of origins such as a skin abscess, urinary tract infection (UTI), bowel perforation, or surgical manipulation of the genital area. The condition primarily affects men between the ages of 50-79 with an incidence of 1.6 per 100,000 in that demographic category. FG has a very high fatality rate with some studies showing 30-80% of patients succumbing to the disease. Therefore, early detection and treatment is crucial. Treatment consists of prompt surgical debridement of the affected tissue and broad-spectrum antibiotics [1,2]. Even with successful initial treatment, serious complications such as urinary, fecal, and cosmetic issues can arise in a large percentage of patients [1].

Prognosis can be determined using Fournier’s Gangrene Severity Index (FGSI), which uses an array of vital signs and lab values to assess the likelihood of survival [3]. Several studies document the validity of FGSI in predicting death in patients with FG. The results have shown that FGSI is a useful prognostic tool for predicting mortality [3]. The authors of a recent study have suggested that diabetes should be added to the scoring system as diabetes continues to be the most common risk factor for FG [4]. Additional metrics such as age, extent of tissue involvement, and comorbidities are included in the Charlson Comorbidity Index (CCI) which has also proven to be a powerful prognostic tool for predicting mortality in patients with FG [5]. The CCI assigns weights to medical comorbidities based on the likelihood of complication or death in medical situations including FG [5,6].

This study explores the potential of using FGSI and CCI for predicting patient outcomes other than mortality, including intensive care unit (ICU) admission and length of hospital stay. Previous studies have assessed the validity of predicting death as a direct outcome of FG, but FG can also cause a wide array of other potential complications. In addition to the above-mentioned scoring systems, this study will consider the diabetic status and the use of glycemic control medications in patients with FG to predict outcomes.

## Materials and methods

This is a multicenter retrospective case series. Inpatient admissions from 2013 to 2023 with the diagnosis of FG were selected from the EPIC Clarity database of the Sisters of Saint Mary hospital system, which consists of a large urban tertiary care center and multiple satellite facilities. The inclusion criteria for this project required a patient to be diagnosed with FG using CPT code N49.3, the necessary lab values to calculate the FGSI score, medical comorbidities to calculate the CCI score, patient demographics, ICU admission, and length of hospital stay. Patients who did not meet all these criteria were excluded from this study. The CPT code N49.3 is a male-specific diagnosis for FG and as a result, all patients in this study were male. These data were extracted from the selected admissions and analyzed.

Generalized linear regression and logistic regression were used for multivariate analysis of continuous and binary outcomes, respectively. The covariates in both models were diabetes status, insulin use, FGSI score, and CCI score. P-values of less than or equal to 0.05 were considered significant.

## Results

This study identified 264 patients who were diagnosed with FG during the selected time period.

Table 1 displays the baseline characteristics of patients diagnosed with FG. Of the cohort, 44 patients died from FG, resulting in a mortality rate of 16.7%. The cohort was entirely male with a mean age of 54.7 years old and an average body mass index of 36.1. The mean FGSI score was 13.2 and the median length of hospital stay was 10 days. Diabetic patients made up 62.1% of the cohort, with 45.8% taking insulin medication and 4.9% taking sodium-glucose cotransporter 2 (SGLT-2) inhibitors. The median CCI score was 1 out of a maximum of 24. Of the cohort, 48.1% required a stay in the ICU.

**Table 1:** Baseline characteristics of patients diagnosed with Fournier's gangrene (N = 264) N, Number; SD, Standard Deviation; SGLT-2, Sodium-Glucose Contransporter-2; FGSI, Fournier's Gangrene Severity Index; CCI, Charlson Comorbidity Index; ICU, Intensive Care Unit

Total (N = 264)			
Baseline Characteristics	Mean ± SD		
Age	54.7 ± 14.1		
Body mass index	36.1 ± 10.6		
	Yes (%)		No (%)
Diabetes status	164 (62.1)		100 (37.9)
Patient taking insulin	121 (45.8)		143 (54.2)
Patient taking SGLT-2 inhibitor	13 (4.9)		251 (95.1)
Vitals	Highest (Mean ± SD)		Lowest (Mean ± SD)
Temperature	38.2 ± 0.8		35.8 ± 2
Respiration rate	32.1 ± 1.1		8.5 ± 5.1
Pulse	121.1 ± 21.4		57.6 ± 17.7
Lab Values	Highest (Mean ± SD)		Lowest (Mean ± SD)
Bicarbonate	28.1 ± 3.9		20.2 ± 4.6
Creatinine	2.1 ± 2.6		1.0 ± 1.0
Hematocrit	38.8 ± 6.1		29.8 ± 6.9
Leukocytes	20.9 ± 9.1		8.8 ± 3.6
Sodium	140.7 ± 4.2		132 ± 4.5
Potassium	4.6 ± 0.6		3.5 ± 0.4
Prognostic Scores	Mean ± SD		
FGSI score	13.2 ± 6.4		
	Median (Interquartile Range)	Minimum, Maximum	
CCI score	1 (1, 3)	0, 15	
Outcomes	Yes (%)	No (%)	
Mortality	44 (16.7)	220 (83.3)	
ICU admission	127 (48.1)	137 (51.9)	
	Median (Interquartile Range)	Minimum, Maximum	
Length of hospital stay	10 (5, 16)	1, 181	

Table 2 shows the association of selected risk factors with mortality and ICU admission. Diabetic patients with FG were not at an increased risk for either of these outcomes compared to non-diabetic patients, regardless of their use of insulin medication. Increased FGSI scores showed a very significant association with both mortality and ICU admission. An increased CCI score had a statistically significant association with mortality but did not show a significant correlation with ICU admission.

**Table 2:** Associated risk factors for mortality and intensive care unit admission ICU, Intensive Care Unit; OR, Odds Ratio; CI, Confidence Interval; FGSI, Fournier's Gangrene Severity Index; CCI, Charlson Comorbidity Index

Patient Factor	Mortality		ICU Admission	
	OR (95% CI)	p-value	OR (95% CI)	p-value
Diabetes status	1.1 (0.6, 2.1)	0.82	1.1 (0.7, 1.8)	0.77
Patient taking insulin	1.5 (0.8, 2.9)	0.21	1.34 (0.8, 2.2)	0.24
Increased FGSI score	1.14 (1.1, 1.2)	< .0001	1.3 (1.2, 1.4)	< .0001
Increased CCI score	1.2 (1.1, 1.4)	0.003	1.1, (1, 1.2)	0.15

Table 3 represents a linear regression model predicting the length of hospital stay for patients with selected risk factors. Whether or not a patient had diabetes or was taking insulin did not have a statistically significant impact on the length of hospital stay. However, a single-point increase in a patient’s FGSI or CCI score had a significant impact on their length of hospital stay.

**Table 3:** Patient risk factors affecting length of hospital stay CI, Confidence Interval; FGSI, Fournier's Gangrene Severity Index; CCI, Charlson Comorbidity Index

Factor Affecting Length of Hospital Stay	Increased Length of Hospital Stay in Days - β (95% CI)	p-value
Diabetes status	0.7 (-3.3, 4.7)	0.71
Patient taking insulin	2.9 (-1, 6.7)	0.15
One-unit increase in FGSI score	1.1 (0.9, 1.4)	< .0001
One-unit increase in CCI score	0.9 (0.03, 1.7)	0.04

## Discussion

This study found that diabetes status was not associated with any of the measured outcomes for patients with FG. Previous studies have shown that diabetes is a major risk factor for a patient developing FG [4], but these results indicate that diabetic patients with FG do not have an increased risk of more serious disease. These findings are consistent with results from other research in the literature that came to similar conclusions showing that diabetes status did not affect mortality in FG patients [7,8]. Insulin is required for type 1 and refractory type 2 diabetes; thus, it is often an indicator of more severe diabetes [9]. This study also failed to show an association between the use of insulin and outcome. These results suggest that appropriate glycemic control through the use of medications during treatment is more important than a history of diabetes.

Diabetes can lead to excess glucose in the urine, which increases the risk of UTI [10]. Underlying UTI is a common initiating event for FG [4]. SGLT-2 inhibitors further increase glycosuria as a means of eliminating blood glucose to improve glycemic control [10]. Research has shown an association between the use of these medications in diabetic patients and the development of FG [11]. While only a small percentage of the patients in this study were taking SGLT-2 inhibitors, these patients did not show an increased association with any of the measured outcomes. This is further evidence that risk factors for contracting FG do not necessarily lead to worse outcomes [7,8].

This study found that the FGSI was useful for predicting health outcomes beyond mortality, including both ICU admission and length of hospital stay. Previous studies have shown the efficacy of using a patient’s FGSI and CCI scores to predict mortality [3,4]. A systematic review and meta-analysis comprising 40 studies with over 2,000 combined patients showed that patients with an increased FGSI score had a significantly increased risk of mortality from the disease. They found that patients with an FGSI score of less than 2 had a mortality rate of just 4.8% compared to those with a score of greater than 2 who had a mortality rate of over 70% (p<0.0001) [12]. Similarly, CCI scores are also associated with mortality in FG, with one study of 144 patients reporting that patients with a decreased CCI score had a significantly reduced risk of death from the disease. The overall mortality rate in this study was 14.6% with a mean CCI score of 2.3 for survivors compared to 5.03 in non-survivors (p<0.001) [13]. While there are more data supporting the validity of FGSI as compared to CCI for predicting mortality in FG patients, both indexes showed significant association across numerous studies [12,13]. Overall, the results from this study, along with those of previous studies, show a stronger association of FGSI with mortality than CCI.

Prediction of outcomes other than mortality helps providers anticipate the needs of complex patients. FG is a highly lethal disease. Physicians need to recognize the patients with the highest risk of complications stemming from FG so they can assign appropriate acuity of care [14]. Accurate prediction of the need for ICU admission facilitates this process. The treatment of FG is also resource-intensive. Studies have shown that the threshold for repeat debridement procedures should be very low to minimize the risk of inadequate source control if the initial procedure fails to eliminate all the affected tissue. Multiple debridements and extended periods of wound care are time-consuming. Accurate prediction of length of hospital stay can help with resource allocation and disposition planning [15]. Several studies have shown the importance of accurately predicting a patient’s length of hospital stay for both resource allocation purposes and overall health outcomes for patients. Many of these studies mention that the lack of accurate systems to predict a patient’s length of hospital stay makes it much more difficult to allocate those resources efficiently and provide optimal care [16]. This study agrees with much of the previous literature on the topic and provides evidence that both FGSI and CCI scores are significantly correlated with the length of hospital stay in patients with FG.

This study appears to be the single largest case series of FG in the current literature and therefore provides a great ability to assess the risk factors associated with poor outcomes related to FG. The outcomes measured in this study found that in addition to overall mortality, ICU admission and length of hospital stay were also correlated with patients’ FGSI and CCI scores on admission. As with all retrospective data, causal relationships cannot be inferred from the observed associations, but the size of this study makes these correlations convincing. Additional limitations to this study involve the fact that all included patients were male. While FG is much more common in males, it can still occur in female patients and therefore more research needs to be conducted to ensure that this data can be reproduced in a more representative population. Also, the lack of information regarding the hemoglobin A1c levels of each diabetic patient who presented with FG made it difficult to truly assess how well each patient’s condition was being controlled. While the medication lists for these patients were available and it was possible to see who was actively treating their diabetes with pharmacological therapy as a proxy, there were no accurate lab values to correlate the use of these medications with good glycemic control. This leaves open the possibility that patients actively taking medication for their diabetes may still have had similar or worse blood sugar levels compared to diabetic patients not using medication to treat their disease.

## Conclusions

The results of this study show that diabetes does not predict a more severe progression of FG. The use of insulin and SGLT-2 inhibitors also failed to predict outcomes. Diabetes is known to be a risk factor for the occurrence of FG, but diabetics respond as well as non-diabetics to treatment. Therefore, it is recommended that diabetic status not be incorporated into the FGSI. FGSI and CCI scores are confirmed as independent predictors of mortality in FG. This study also showed that they can be used to predict the need for ICU admission and length of hospital stay. Of the two scoring systems, FGSI shows a stronger association with outcome. In addition to providing prognosis, FGSI can be a valuable tool to help guide the acuity of care, resource allocation, and disposition planning.
